# Case Report: Cavernous Sinus Syndrome as the Initial Presentation of Multiple Myeloma

**DOI:** 10.3389/fopht.2022.849343

**Published:** 2022-03-30

**Authors:** Rebecca F. Silverman, Lawrence Hanson, Navid Salahi, Zhonghua Li, Marina Boruk, Nickisa M. Hodgson

**Affiliations:** ^1^ Department of Ophthalmology, SUNY Downstate, Brooklyn, NY, United States; ^2^ Department of Pathology, SUNY Downstate, Brooklyn, NY, United States; ^3^ Department of Otolaryngology, SUNY Downstate, Brooklyn, NY, United States

**Keywords:** oncology, neuroophtalmology, cavernous sinus, cranial nerve (CN) disorders, multiple myeloma

## Abstract

Multiple myeloma (MM) is the second most common hematologic malignancy and most common primary bone malignancy. Ocular manifestations of MM are extremely rare and may be the first presentation leading to diagnosis. Ophthalmologists routinely encounter cavernous sinus syndrome, and there is a wide range of possible etiologies. Here, we present a case of a patient presenting with diplopia, ptosis, and ophthalmoplegia found to have a cavernous sinus plasmacytoma with systemic workup consistent with MM. MM is a rare cause of cavernous sinus syndrome and should be considered in the setting of a skull base mass.

## Introduction

Multiple myeloma (MM) is a malignancy characterized by monoclonal proliferation of plasma cells in the bone marrow that can cause lytic lesions, frequent infections, and renal failure or can present asymptomatically (1). Plasmacytomas are malignant plasma cell neoplasms that can occur as solitary disease or as a feature of MM. Solitary plasmacytoma can present as extramedullary plasmacytoma (EMP) or solitary bone plasmacytoma (SBP). Plasmacytomas can also be secondary to MM when associated with systemic disease (2). SBP or EMP cannot be diagnosed without a systemic workup including bone marrow biopsy to rule out MM, and still up to 85% of those with solitary plasmacytoma will eventually develop MM (3).

Periocular involvement of MM is extremely rare, and only around 50 patient reports exist in the literature (4). When there is orbital involvement, the most common presentations are proptosis and periocular swelling, and the ophthalmoplegia or ptosis is more rare (5). Plasmacytoma of the cavernous sinus is even more rare, with only a few reports in the literature. We report a case of a 60-year-old man who presented with diplopia, ptosis, and ophthalmoplegia found to be due to a plasmacytoma of the cavernous sinus associated with concomitant MM.

## Case Presentation

A 60-year-old man with a past medical history of diabetes and hypertension presented to the emergency room with intermittent diplopia and pain above his left eye for 2 weeks. He reported ptosis of the left eye for 1 week. On ophthalmic examination, he reported that the ptosis had been progressively worsening over the last week. Visual acuity was 20/20 in both eyes with normal intraocular pressure. Pupils were notable for left mydriasis, poor reactivity to light, without an afferent pupillary defect. Extraocular movements were notable for a −1 limitation on supraduction and infraduction of the left eye, without deficit on intorsion or external rotation. Sensation in the distribution of the trigeminal nerve was not checked at initial presentation. External exam was notable for ptosis with margin to reflex distance 1 (MRD1) of 2 mm and a mild exotropia of the left eye. Fundus exam was unremarkable.

**Figure 1:**
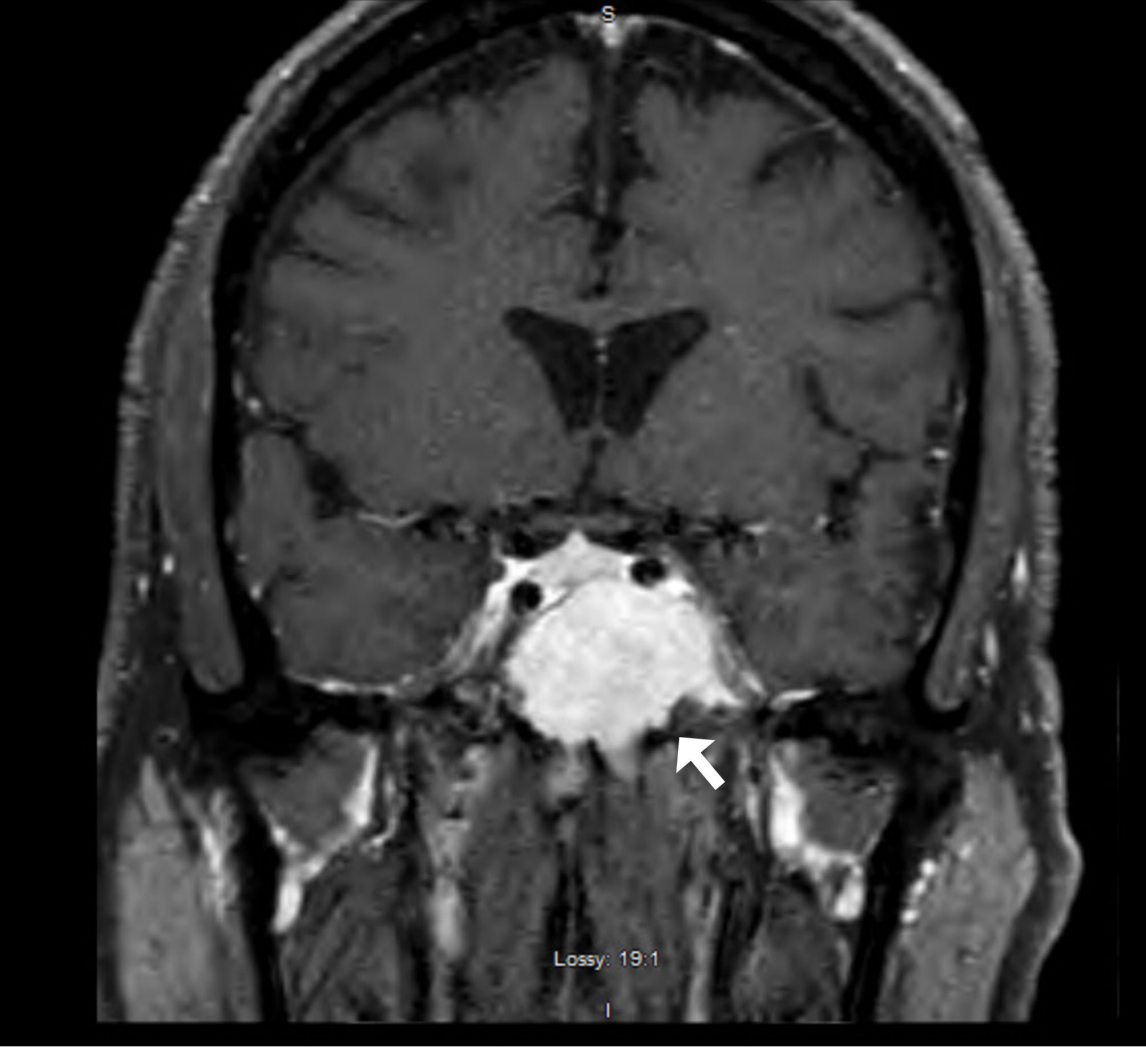
T1-enhanced fat-suppressed coronal MRI demonstrating a large avidly enhancing mass involving skull base with the majority of tumor burden centered in clivus with invasion of left sphenoid sinus and left cavernous sinus.

**Figure 2:**
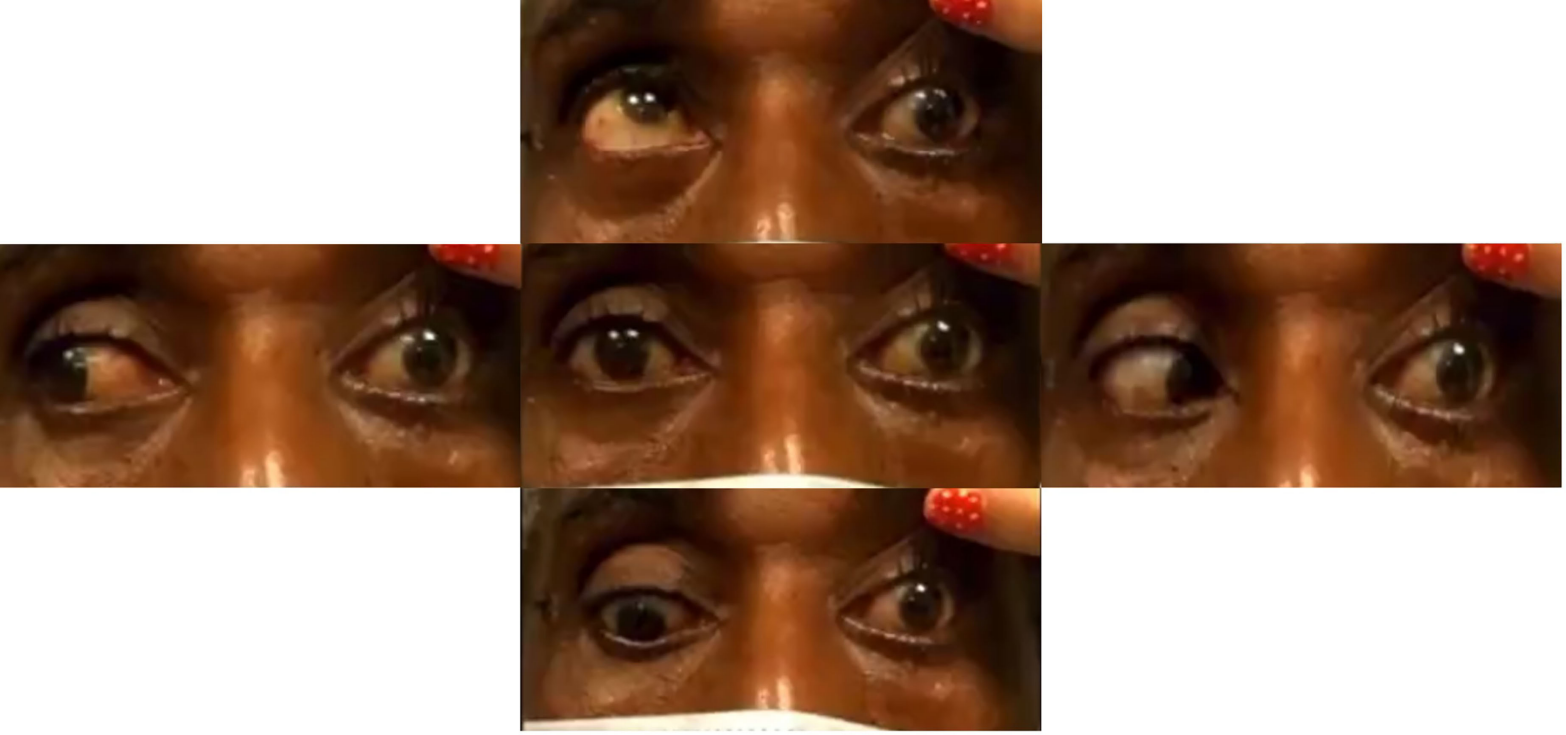
Extraocular movements demonstrating reduced supraduction, infraduction, abduction, and adduction of the left eye with a down and out left eye in primary gaze (center).

**Figure 3:**
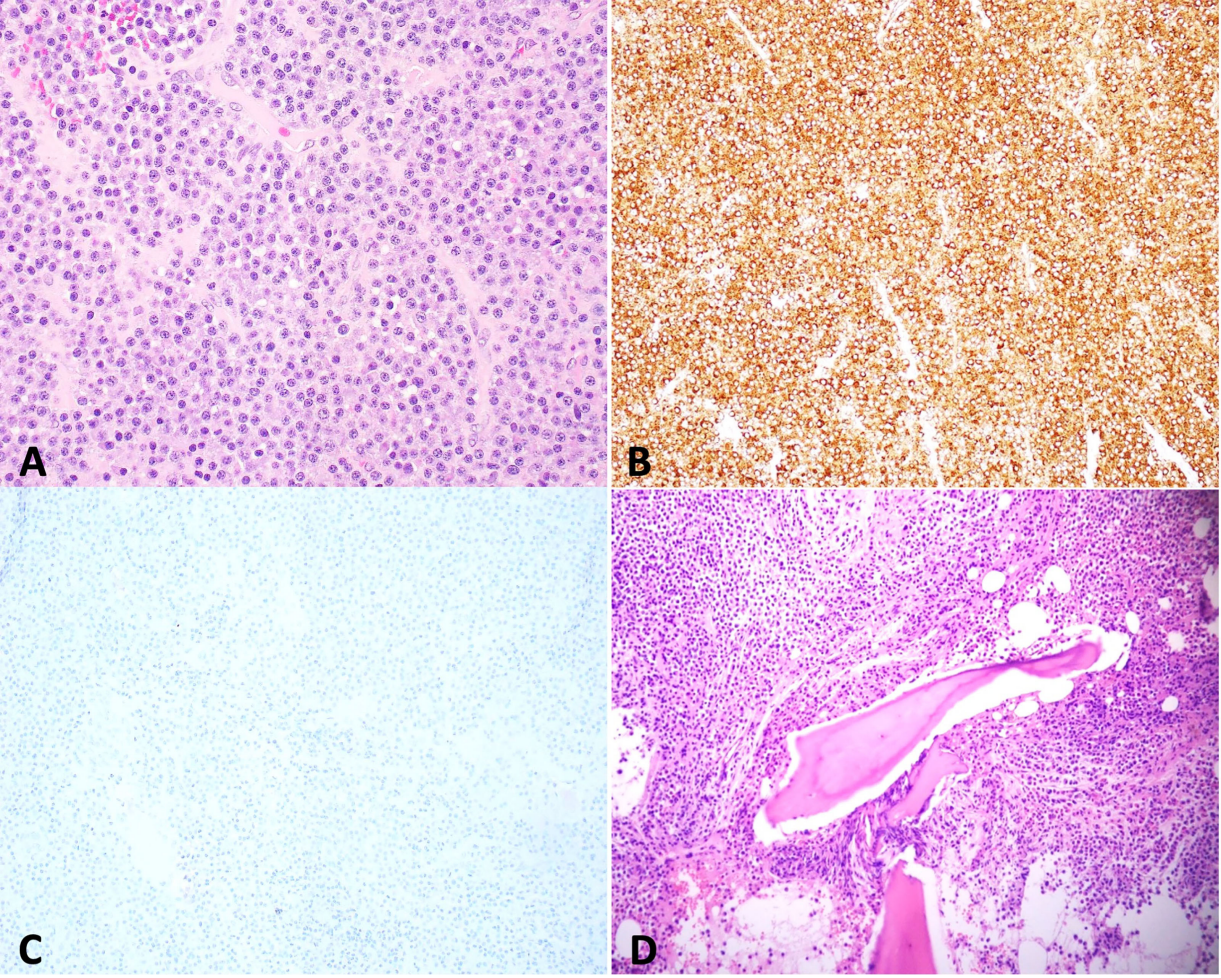
Biopsy of skull base mass shows sheets of plasma cells [**(A)** hematoxylin and eosin stain (H&E), 400×] that are positive for lambda [**(B)** 100×] and negative for kappa [**(C)** 100×] by immunohistochemistry, indicating lambda light-chain restriction. Bone marrow biopsy reveals that sheets of plasma cells infiltrate in bone marrow [**(D)** H&E, 200×] with lambda light-chain restriction (date not shown).

A CT head revealed a 3.2 × 3.3cm left clival mass with cavernous sinus and sphenoid sinus extension and associated osseous erosion. An MRI was performed, which demonstrated a 3.5-cm enhancing destructive mass within the clivus extending into the left cavernous sinus and partially encasing the left cavernous internal carotid artery (**Figure 1**). Smaller enhancing lytic skull base lesions were also noted. The patient initially refused biopsy of the lesion and his clinical examination rapidly worsened. Four days after initial examination, he developed complete ptosis with a fixed and dilated pupil. His motility worsened with inability to supraduct, infraduct, and adduct and −3 abduction (**Figure 2**). He underwent repeat imaging with MRI brain and orbits, which did not show interval change in size of lesion. Endoscopic biopsy at that time of the mass involving the sphenoid sinus was consistent with lambda light-chain restricted plasmacytoma (**Figure 3**).

Systemic workup was performed with skeletal survey, demonstrating multiple lytic and destructive bone lesions throughout the pelvis, multiple vertebral bodies, and bilateral proximal femurs. Serum protein electrophoresis was notable for an M-spike of 3.8g, with an Immunoglobulin G (IgG) prevalence and elevated lambda light chains. Bone marrow biopsy was performed, which demonstrated >10% plasma cells, found to be IgG lambda light-chain restricted MM with high-risk cytogenetics, FGFR3-IGH gene rearrangement [t(4;14) translocation] (**Figure 3**).

He was started on pulse steroids with dexamethasone for 4 days and then received radiation therapy to the base of the skull at 3,500 cGy. He was discharged on a dexamethasone taper with plans to start systemic therapy. He was seen in our eye clinic 1 week after skull-based radiation therapy and, unfortunately, had no improvement in his clinical examination. The initiation of chemotherapy was delayed due to COVID-19 infection and subsequent failure to thrive. After recovery from COVID-19, the plan was to begin bortezomib; however, the patient developed hypercalcemia and altered mental status after the first dose. Imaging showed worsening lesions of the thoracic spine with cord compression. The decision was then made to stop chemotherapy and pursue palliative care measures. Unfortunately, the patient expired shortly thereafter, just 5 months after diagnosis.

## Discussion

MM is a systemic disease that is characterized by a monoclonal proliferation of plasma cells that are detectable in the serum or urine (6). It effects 7.1 per 100,000 men and women per year in the United States with a median age at diagnosis of 69 years old (7). With localized disease the 5-year survival rate is 77.5%; however, the 5-year survival rate drops to 54.5% when there is distant spread (7).

In a healthy individual, plasma cells are produced from B cells that produce immunoglobulins. Each immunoglobulin contains a heavy chain and two light chains. The five types of heavy chains are IgG, IgM, IgA, IgE, or IgD (8). Light chains can be kappa or lambda. Most commonly in MM, there is preponderance of IgM or IgG. As MM continues to progress, light chains will be produced at a greater rate than heavy chains, which can be useful in monitoring disease progression. Screening includes measuring serum and urine protein electrophoresis, immunofixation studies in serum and urine, and detection of immunoglobulin free light chains. In patients where serum and urine testing is suspicious for myeloma, bone marrow biopsy should be performed. Diagnosis of MM requires at least 10% of plasma cells in the bone marrow or biopsy-proven bony or EMP, plus the presence of one myeloma defining event: hypercalcemia, renal insufficiency, anemia, or osteolytic bone lesions (9).

Plasmacytomas are histologically the same as MM, with tumors showing predominantly plasma cells that stain positively for CD38 and CD138 with the majority having immunoglobulin light-chain restriction (4). Plasmacytomas typically begin in the bone but can also found in soft tissue and are then designated extramedullary (2). SBP or solitary EMP has a better prognosis than MM alone; however, many will eventually develop MM (10).

In MM, the most common manifestations on presentation are anemia, infections, lytic bone lesions, or renal insufficency (6). Periocular involvement in MM is extremely rare and can be due to local tumor growth or hematologic abnormalities due to the underlying plasma myeloma and, therefore, can affect virtually any part of the eye. Intraocular manifestations that have been reported include uveal cysts, corneal crystals, exudative macular detachments, vascular occlusions from hyperviscosity syndrome, and retinal hemorrhages or cotton wool spots (11). Many of these findings, however, may be asymptomatic or found on autopsy (12). Orbital involvement of MM has also been reported. In a review, Burkat et al. found that, of 52 patients with orbital involvement, 35% of had orbital symptoms as their initial presentation of their disease (10).

Cavernous sinus involvement, however, is more rare and has been reported in only case reports and series (13–28). The majority of cases reported are in patients with known diagnoses of MM or EMP (15, 20, 21). Of these case reports, there were only three patients whom initially presented with cavernous sinus syndrome that eventually lead to a diagnosis of MM. Each of these three cases had varied clinical presentations. Ko et al. reported a 48-year old-male who presented with vertigo, diplopia, and intermittent left retro-orbital pain for 1 week and was found to have left abducens palsy with left V2 hypesthesia. The patient was initiated on corticosteroids before imaging was obtained for presumed Tolosa-Hunt Syndrome. Eventually, imaging and laboratory work confirmed a cavernous sinus mass and a diagnosis of MM. The mass was removed and histological examination confirmed plasmacytoma. Galea et al. reported a 58-year-old female patient who presented with vertical diplopia and variable left upper lid ptosis. Examination was notable for limitation of supraduction that worsened on left gaze. This patient was initiated on chemotherapy without mention of radiation to the skull base lesion. Last, Lam et al. reported a 76-year-old male patient who presented with diplopia and was found to have 1 mm of right ptosis, limitation of adduction, elevation, and depression of the right eye with right V1 hypesthesia. Imaging was nonconclusive without evidence of a mass. The patient was treated only with corticosteroids. Given the aggressive nature of the disease, the patient died 3 months after presentation. An autopsy revealed that the cavernous sinus was filled with malignant plasma cells. These patients presented similarly to the present case with varied cranial nerve involvement.

Cavernous sinus syndrome often presents with multiple cranial nerve palsies. Prompt imaging must be performed to rule out life-threatening diagnoses including tumor, aneurysms, or arteriovenous fistulas. Our case highlights the importance of histological examination of tumors involving the cavernous sinus when tissue can be safely taken, as the diagnosis of plasmacytoma lead to discovering systemic MM in this patient. In this case, the mass extended into the sphenoid sinus, providing a safer biopsy location. Plasmacytoma must be included in the differential diagnosis of any cavernous sinus mass, contributing to multiple cranial nerve palsies. These tumors can progress rapidly as in our patient who had worsening ophthalmoplegia and ptosis in just 4 days during admission. In a case reported by Lam et al., similarly. the patient’s clinical exam worsened rapidly over 2 weeks even with steroid initiation (15). This potential for rapid decline has not been well documented and highlights the importance of prompt diagnosis and treatment. Treatment of plasmacytoma is typically with radiotherapy of the mass, with chemotherapy in conjunction for systemic myeloma. There have been reports suggesting that adjuvant chemotherapy should be considered even in solitary EMP (26). This is in contrast to other tumors of the clival region in which neurosurgical resection would be mandatory, highlighting again the importance of biopsy in plasmacytoma.

Our case highlights a rare presentation of MM. There have been very few reports in the literature demonstrating cranial nerve palsies as presenting clinical features of MM. Few of these reports have been in the ophthalmic literature. Plasmacytomas and MM can be asymptomatic until spread is more severe; therefore, it is important that ophthalmologists are aware of the way in which plasmacytomas can present in the orbit and periocular region. Plasmacytoma is important to consider on the differential diagnosis of any space occupying lesion involving the orbit and cavernous sinus. A multidisciplinary approach is of the upmost importance in ensuring appropriate and timely diagnosis and treatment.

## Data Availability Statement

The original contributions presented in the study are included in the article/supplementary material. Further inquiries can be directed to the corresponding author.

## Ethics Statement

Written informed consent was obtained from the relevant individual for the publication of any potentially identifiable images or data included in this article.

## Author Contributions

All authors contributed to manuscript writing and revision and read and approved the submitted version.

## Conflict of Interest

The authors declare that the research was conducted in the absence of any commercial or financial relationships that could be construed as a potential conflict of interest.

## Publisher’s Note

All claims expressed in this article are solely those of the authors and do not necessarily represent those of their affiliated organizations, or those of the publisher, the editors and the reviewers. Any product that may be evaluated in this article, or claim that may be made by its manufacturer, is not guaranteed or endorsed by the publisher.
